# Evolution, Interactions, and Biological Networks

**DOI:** 10.1371/journal.pbio.0050011

**Published:** 2007-01-16

**Authors:** Joshua S Weitz, Philip N Benfey, Ned S Wingreen

## Abstract

Shifting the perspective of the questions we ask will ensure that network theory continues to excite the network theorists, but more importantly, that it remains vital to progress in biological research.

The study of networks has expanded rapidly over the last 10 years; networks are now widely recognized not only as outcomes of complex interactions, but as key determinants of structure, function, and dynamics in systems that span the biological, physical, and social sciences [1–4]. The “new science of networks” [5] has introduced novel paradigms of systems behavior, including small-world structure [6], scale-free networks [7], and the importance of modularity [8] and motifs [9]. Some of these ideas have been transplanted into biology, and the results thus far are mixed but promising. Certainly, the study of biological networks has brought new opportunities for publication, yet much effort has been placed at discovering particular patterns in unexpected places—e.g., scale-free distributions in gene regulatory networks [10]—and these findings come with the caveat that similar patterns do not necessarily point to a common mechanistic origin [11]. Despite the many findings of power laws and hubs in biological systems, it is important to keep in mind that in biology, networks are not of interest solely (even primarily) for their abstract properties. So, if biologists working at the bench or in the field remain skeptical of what the study of networks can do for them and for their discipline, network scientists should not be surprised. These biologists want to know: what makes biological networks distinct and why should non-networkologists care?

As Dobzhansky famously noted, nothing in biology makes sense except in the light of evolution [12]. This is particularly true of biological networks, and we believe that the lens of evolution provides an exciting opportunity to link disciplines in ways that address fundamental challenges in biology. When mathematicians and physicists discuss the “evolution” of a network, they are often describing the dynamics by which a particular network structure grows and changes [13]. When biologists discuss the evolution of networks, they typically mean that fitness is network-dependent and selection acts to optimize across a landscape of networks [14]. Both definitions are useful, indeed complementary; the former focuses attention on possible dynamical origins for network structure [11] and the latter highlights the possibility that higher-level properties resulting from networks may be selected for [15,16]. Here we offer a third way to think about networks.

The central organizing principle in the study of networks is that interactions between elements in a complex system are heterogeneous. Some elements are connected to many others, some to very few, and interaction strengths and dynamics may vary widely. This is certainly true of the vast majority of biological systems. A primary consequence of these heterogeneous interactions is that patterns and properties emerge at different scales of organization from the interactions themselves. What is distinct about biological networks is that they arise as a result of evolution, with selection operating at the level of individuals and as a result of interactions between organisms.

We propose to think about networks within organisms as complex phenotypes interacting with other networks. When two organisms and their respective networks interact, the outcome at multiple scales will reflect game-theoretic and density-dependent interactions [17–19]. Further, such an approach provides a framework for assessing how higher-order properties (e.g., robustness or resistance to attack) may emerge under constraints imposed by other organisms. We focus our attention on three types of networks within organisms—regulatory networks, sensory networks, and resource delivery networks—and we leave aside the evolution of networks of organisms (e.g., syntrophic networks or food webs) for which the concepts of game theory and density dependence are already essential tools of analysis [20]. Our choice of examples takes aim at a central question in biology: how do organisms evolve and maintain complex and diverse functions?

To begin, consider the regulatory network of a temperate phage. Once inside a bacterial cell, a phage coopts its host's machinery and begins to modulate a system of promoters and pathways leading to cell lysis or integration [21,22]. Co-infections may occur, in which case another phage with a related but genetically distinct encoding of a regulatory network may be present. Networks that can function “optimally” in isolation may perform poorly (or be subject to exploitation) when mixed with competitors, as is the case of defective interfering particles [23]. Competition among regulatory networks may lead to selection for robustness, the development of strain immunity, or altered host control.

Sensory networks provide another example. Systems biology is only beginning to explore the strategies used by cells to function reliably using noisy machinery. Some emerging themes include digital logic, integral feedback, and limit cycles [24]. These paradigms are representative of systems that are intrinsically insensitive to noise. However, these paradigms do not address the unique challenge of accurately sensing environmental signals: namely that real changes in the signal must be reliably distinguished from fluctuations in the levels of network components. Further, these paradigms do not address how individual cells cope with the exchange of signaling molecules produced by other individuals that may be trying to regulate or maintain function in the environment or may be attempting to disrupt intentionally the function of other individuals. If we want to know how cells reliably integrate information from multiple signals, we should also be concerned with fluctuations induced exogenously by the presence of alternative networks, some operating with the same or similar signaling molecules, and some actively interfering with signaling.

Finally, consider physical delivery networks such as the root system of a plant or the branching structure of a tree. Both networks must provide structural support, facilitate the delivery of nutrients and water from soil to shoot, confer resistance against catastrophic embolisms, all while scaling up their components and connectivity from year to year [25]. Yet a tree will have diminished reproductive success if its branching/root structure confers enhanced functioning in isolation, but the structure is easily shaded out above ground or is out-competed below ground by the network of an adjacent tree.

Evaluating and searching for optimal network design involves more than just finding peaks in a fitness function. The suitability of a given network design must be considered in the context of alternatives. Networks in this light can be seen as strategies, in much the same way that rapid growth or efficient growth are alternative strategies for organisms competing for a common resource. Unlike peaks in a fitness function, the success of a strategy depends on how well it can out-compete other strategies when it is rare, as well as how well it can resist invasion by other strategies when it is common. Coexistence of multiple network structures in biological systems may well reflect these types of game-theoretic interactions.

If we are to develop an evolutionary ecology of networks then we should: (i) improve classification schemes for describing the microstates of networks; (ii) develop a more rigorous, and perhaps, system-specific understanding of permitted moves and trade-offs between networks; and (iii) use the principles of game theory and adaptive dynamics to consider how networks interact via their emergent properties. For regulatory networks, do features emerge primarily through gene duplication with subsequent neofunctionalization, what are the fitness and energetic costs of such duplication events, or are there other more complex processes at work [26,27]? For sensory networks, tradeoffs may involve limitation of the number or production of pathway components and therefore may be an implicit constraint to adding additional signaling cascades to sense distinct conditions/molecules. The study of resource delivery networks raises the question of allocation strategies when network components involve fixed costs, such as the investment of tissue and energy [28]. In all cases, we are confronted with a substantial challenge for theory: what is a meaningful level of granularity with which to describe a network that itself is a vast simplification of complex interactions?

Ecologists have long advocated the study of how interactions among individuals lead to ecosystem-level networks that, in turn, shape community assembly, stability, and robustness [20]. The availability of high-throughput data in molecular and systems biology suggests new opportunities for cross-disciplinary synthesis. What biological or ecological function does a network perform or mediate? How robust is network-associated function with respect to various types of noise? How does network structure influence and reflect the process of evolution? To answer these questions, it may prove essential to consider how organisms with a given type of network invade a system dominated by individuals of a given type or of a coalition of types, and if so, what systems-level properties emerge. Shifting the perspective of the questions we ask (and the framework in which we ask them) will ensure that network theory continues to play an integral role in furthering biological research.
